# An Overview of Appetite-Regulatory Peptides in Addiction Processes; From Bench to Bed Side

**DOI:** 10.3389/fnins.2021.774050

**Published:** 2021-12-09

**Authors:** Olesya T. Shevchouk, Maximilian Tufvesson-Alm, Elisabet Jerlhag

**Affiliations:** Department of Pharmacology, Institute of Neuroscience and Physiology, The Sahlgrenska Academy at the University of Gothenburg, Gothenburg, Sweden

**Keywords:** gut-brain axis, reward, dopamine, ghrelin, GLP-1, amylin, addiction, mental health

## Abstract

There is a substantial need for new pharmacological treatments of addiction, and appetite-regulatory peptides are implied as possible candidates. Appetite regulation is complex and involves anorexigenic hormones such as glucagon-like peptide-1 (GLP-1) and amylin, and orexigenic peptides like ghrelin and all are well-known for their effects on feeding behaviors. This overview will summarize more recent physiological aspects of these peptides, demonstrating that they modulate various aspects of addiction processes. Findings from preclinical, genetic, and experimental clinical studies exploring the association between appetite-regulatory peptides and the acute or chronic effects of addictive drugs will be introduced. Short or long-acting GLP-1 receptor agonists independently attenuate the acute rewarding properties of addictive drugs or reduce the chronic aspects of drugs. Genetic variation of the GLP-1 system is associated with alcohol use disorder. Also, the amylin pathway modulates the acute and chronic behavioral responses to addictive drugs. Ghrelin has been shown to activate reward-related behaviors. Moreover, ghrelin enhances, whereas pharmacological or genetic suppression of the ghrelin receptor attenuates the responses to various addictive drugs. Genetic studies and experimental clinical studies further support the associations between ghrelin and addiction processes. Further studies should explore the mechanisms modulating the ability of appetite-regulatory peptides to reduce addiction, and the effects of combination therapies or different diets on substance use are warranted. In summary, these studies provide evidence that appetite-regulatory peptides modulate reward and addiction processes, and deserve to be investigated as potential treatment target for addiction.

## Introduction

### Overview of Addiction

Addiction/dependence is often referred to as a chronic, relapsing brain disorder which, depending on the drug used, can be divided into alcohol use disorder (AUD) and substance use disorders (SUD; nicotine, amphetamine, cocaine, opioids). These different terms are used interchangeably throughout this overview. In general, addiction develops after the initial acute exposure to an addictive drug followed by more chronic use. It develops as a result of recurring cycles of binge/intoxication followed by withdrawal/negative affect followed by preoccupation/anticipation until the next drug intake session (Koob, 2014). The details of these three phases are described elsewhere (Koob, 2014), but in short, the binge/intoxication involve reward/reinforcement, the withdrawal/negative effects include abstinence symptoms specific to the drug used, and the preoccupation/anticipation is characterized by craving induced by the drug, a cue or stress. These repeated cycles, together with the fluctuating doses of the addictive drugs in the brain, lead to long-term neuroadaptations; where this final stage is referred to as addiction. These neuroadaptations, in turn, alter the individuals' behaviors, including motivation, emotion, and decision-making. Furthermore, they change the responses of the brain toward the drug being used in a multitude of ways.

**Figure 1:**
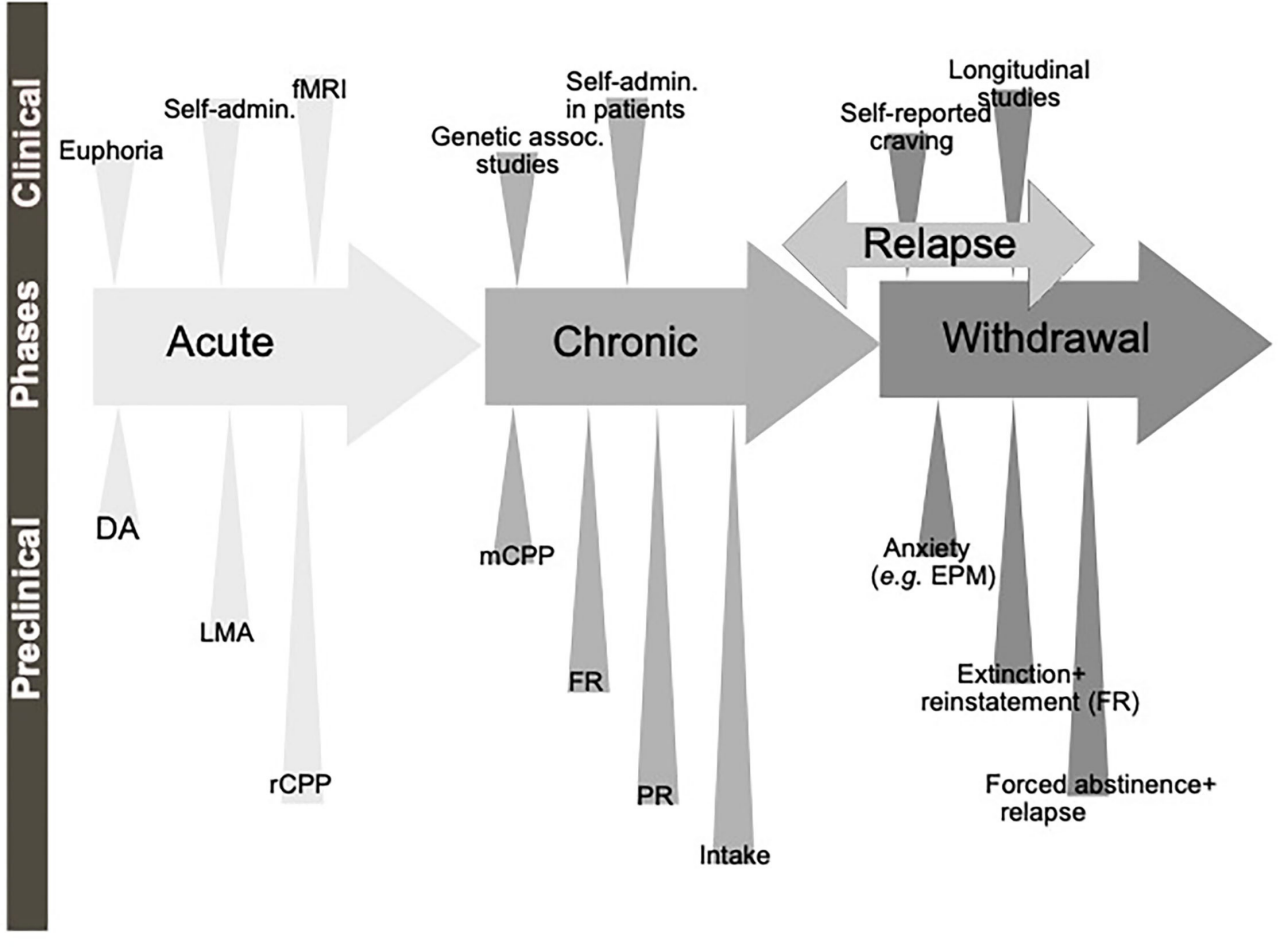
Schematic illustration of the different phases of the addiction cycle. Alcohol or drugs of abuse when consumed acutely can be modeled both in animals (preclinical) and humans (clinical models). Similarly, when exposed for longer periods of time other models can be applied. During withdrawal and relapse other behaviors are evident. Dopamine release in nucleus accumbens (DA), locomotor stimulation (LMA), reward of conditioned place preference (rCPP), memory of alcohol reward in the CPP test (mCPP), motivation (FR, and PR), Alcohol and drug intake (Intake), functional magnetic resonance (fMRI).

A diagnosis of AUD/SUD includes behaviors like loss of control over intake, craving and impaired cognitive functioning, and impaired functions like negative social consequences, physical tolerance, and withdrawal symptoms (Jayaram-Lindström et al., 2016). The findings that individuals with heightened alcohol reward/stimulation at youth display a higher risk of an AUD diagnosis (King et al., 2021), further highlight the importance of evaluating the neurobiological alterations observed during the binge/intoxication phase. The biological processes and neurocircuits of importance for the acute and chronic effects of a drug may to some extent overlap but may also diverge. Therefore, it is important to separately study the effect of treatments during early drug experiences, which we will call here acute effects of substance use, versus the effects after a prolonged period of substance use, which we will call chronic effects (Figure 1).

Although all these components of the addiction cycle are presented in this overview (Figure 1), due to design limitations, some of them are challenging to study in one model. Indeed, one animal model cannot study all these different stages and various different preclinical models reflecting different aspects exist, and these are summarized in Table 1. Moreover, clinical studies can also be used to model different aspects of the addiction cycle in humans (Table 1). When combined, these models can more accurately study the complex mechanisms important for the acute and chronic effects of a drug (for review Sanchis-Segura and Spanagel, 2006).

**Table 1:** Descriptions of the various models associated with the different phases of the addiction cycle.

**Phase of SUD**	**Species**	**Model of a component of addiction**	**Description**
Acute	Mouse	Locomotor activation (LMA)	Most drugs increase motor activity, which correlates with dopamine release and reward. Treatments that reduce drug intake and reward tend to also reduce the LMA following an acute dose of the addictive drug
Acute, chronic	Mouse	Locomotor sensitization (LMS)	When a drug is administered multiple times, the magnitude of locomtor activation increases each time, indicating the brain is getting sensitized to the drug
Acute	Mouse/Rat	(Mesolimbic) dopamine (DA) release	The release of dopamine from VTA to NAc shell is the most studied and most robust neurochemical correlate of reward (positive affect) gained from addictive drugs, though DA signaling in other regions can also be relevant
Acute, chronic	Mouse, human	Head scratching/twitch in mice, self-reported euphoria in humans	Difficult to operationalize, the head-twitch response in mice is behavioral model of hallucinogen effects
Acute	Mouse	Conditioned place preference (CPP)	After repeatedly pairing a chamber with distinct sensory features to a drug and another distinc chamber to vehicle, the preference for the two chambers is assessed. Treatments to decrease reward of the drug are administered simultaenously to conditioning
Acute	Rat	Intracranial stimulation threshold	Individual subjects respond for rewarding electrical self-stimulation at a certain rate until their reward threshold, addictive drugs tend to decrease this threshold
Chronic	Mouse	Memory CPP	Similar to CPP above, here the treatment to decrease reward is administered during the post-conditioning test, to evaluate whether the treatment can disrupt the already formed positive reward memory formed during the conditioning
Chronic, withdrawal	Rat, vervet monkey	Intermittent access two-bottle choice (i.a. TBC); forced abstinence followed by reintroduction	A bottle of water and a bottle of (10-20%) ethanol is available in the homecage on an intermittent basis, intake and alcohol preference over water is measured, post-withdrawal relapse drinking is usually higher than before withdrawal
Chronic, withdrawal	Rat, mouse, human	Self-administration (oral or iv), also called operant conditioning - Fixed ratio (FR) - Progressive ratio (PR) - Extinction - Drug or cue-triggered reinstatement	The subject is taught to press a lever, button or perform a nose-poke in order to receive a dose of drug orally or iv. In FR (e.g., FR1, FR3, etc) the number of responses per administered dose is constant (e.g., 1 press, 3 presses, etc). In PR, the number of responses necessary increases over the session (e.g., 1, 2, 4, 9, etc) – this is considered a better test of motivation as the animal needs to work increasingly hard to receive the reward. During extinction, the subject is placed in same environment as for conditioning, but their responses fail to produce any reward. Following from extinction, rapid reinstatement is achieved by triggering with a low dose of the drug or a cue previously associated with the drug reward
Withdrawal	Rat, mouse	Abstinence symptoms such as anxiety measured by Elevated plus maze (EPM)	Withdrawal symptoms include an increase of anxiety, this can be tested by allowing the animal to explore a maze with some closed arms (dark, less anxiogenic) and some open arms (light, more anxiogenic)
Chronic	Human	Genetic associations	Polymorphisms of genes related to the studied signaling systems can be correlated to prevalence of addiction or drug self-administration responses
Chronic	Human	Human laboratory studies/ Clinical studies	For approved drugs, patients already taking a treatment can be interviewed or included in a controlled study to measure their drug intake

**Figure 2:**
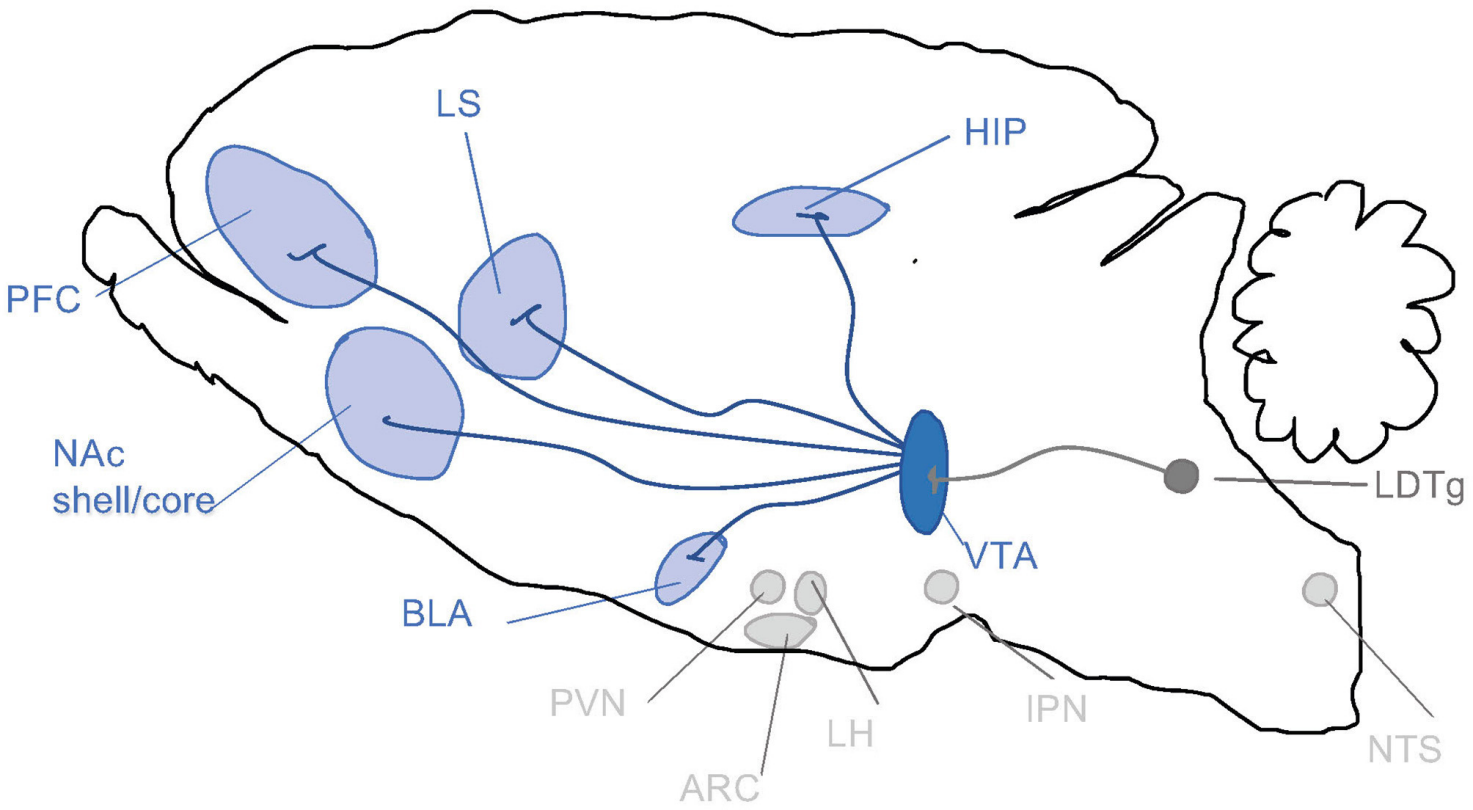
Schematic illustrations of some selected brain regions central for addictions. Dopamine neurons of the ventral tegmental area (VTA) project (dark blue) to brain regions such as prefrontal cortex (PFC), nucleus accumbens (NAc) subdivisions core and shell, basolateral amygdala (BLA), lateral septum (LS) and hippocampus (HIP). Alcohol and other drugs of abuse dopamine release in such areas, which is associated with reward/stimulation/euphoria. Several of these areas are targeted by nucleus tractus solitarii (NTS) of the brain stem. Both the VTA and NAc shell are targeted by the projections from laterodorsal tegmental area (LDTg), which to some extent are cholinergic (dark gray). Another selected area of interest include paraventricular nucleus (PVN), arcuate nucleus (ARC) and lateral hypothalamus (LH) and interpeduncular nucleus (IPN).

When it comes to the above-mentioned neuroadaptations, they include various brain regions and signaling systems of importance. Preclinical and clinical studies pinpoint the mesolimbic dopamine system as a central neurocircuitry for addiction processes (Figure 2). It consists of dopamine neurons of the ventral tegmental area (VTA) projecting to the nucleus accumbens (NAc) shell, amygdala, prefrontal cortex and hippocampus. Moreover, neuronal projections targeting the mesolimbic dopamine system modulate the activity thereof and may thus be important for addiction processes. This includes, but is not limited to, projections from the nucleus of the solitary tract (NTS) or cholinergic projection from the laterodorsal tegmental area (LDTg) onto the VTA. It is well established in both man and animals that alcohol and other additive drugs enhanced dopamine in NAc shell, associated with euphoria, and that addiction is associated with a reduced number of dopamine receptors in this area (Jayaram-Lindström et al., 2016). It should however be emphasized that other brain regions and neurotransmitters are also important for behaviors as complex as addiction.

In attempts to develop novel treatments of addiction, the mechanisms important for the addiction processes should be elucidated. Although complex, both preclinical and clinical studies suggest that appetite-regulatory peptides are essential, even if primarily modulatory; the focus of the present overview.

### Overview of Appetite-Regulatory Peptides

Our appetite is modulated *via* various pathways, where satiety hormones like cholecystokinin, polypeptide YY, neuropeptide Y, melanin concentrated hormone, leptin, neuromedin U, galanin amylin and glucagon-like peptide-1 (GLP-1) and the orexigenic peptide ghrelin play dominant roles (Crooks et al., 2021). Although crucial for hedonic and homeostatic feeding, these appetite-regulatory peptides modulate other physiological properties, including addiction processes. We argue that GLP-1, amylin and ghrelin are of extra interest as pharmaceuticals of GLP-1/amylin are approved for obesity and/or diabetes and ghrelin is the predominant feeding-enhancing hormone. Therefore, the present overview describes if and how GLP-1, amylin or ghrelin modulate reward, reinforcement and addiction processes.

GLP-1 is a peptide produced from its precursor preproglucagon in both the gut and the NTS in the hindbrain, that slows down digestion, suppresses appetite and enhances glucose-stimulated insulin release. When it comes to feeding, GLP-1 reduces the homeostatic and hedonic aspects thereof. Gut-produced GLP-1 acts locally on vagal afferents that project to the brainstem, but it is also released into the pancreas and general circulation. Within the brain the primary production site is NTS, however some GLP-1 is also produced in the medullary reticular formation and the olfactory bulb (Merchenthaler et al., 1999). The NTS projects to many brain regions associated with reward, memory, emotion and motivation and the same brain regions also express GLP-1 receptors (GLP-1R) (Merchenthaler et al., 1999; Graham et al., 2020). In blood, GLP-1 is rapidly degraded by dipeptidyl peptidase 4 (DPP-4) and few if any of circulating GLP-1 enters the brain before degradation. To counteract this short duration of GLP-1, synthetic longer-acting agonists more resistant to DPP-4 degradation have been developed. Multiple GLP-1R agonists have been approved for treating type 2 diabetes (T2D) and/or obesity (see Kalra et al., 2021, for a recent review). The agonists most relevant for this overview are exendin-4 (Ex-4), liraglutide, dulaglutide, semaglutide. The former two are injected daily or twice daily, while the latter is injected weekly, with semaglutide being the first available oral formulation. Besides GLP-1R agonists, multiple inhibitors of DPP-4, such as sitagliptin and linagliptin, are used to treat T2D as they enhance the endogenous levels of GLP-1. In addition, not clinically approved but widely used in research is one antagonist; Exendin-9-39 (Ex-9).

The β-cells of the pancreas produce and secrete amylin following stimuli like nutrients or meal initiation (for review see Lutz and Meyer, 2015). Amylin is well-known for its ability to control glucose homeostasis, and one compound acting on the amylin pathway is the FDA approved pramlintide, which is used by patients with diabetes type I (for review see Lutz and Meyer, 2015). The additional physiological properties of amylin are vast and include a reduction in appetite, feeding and body weight (for review see Lutz and Meyer, 2015). The reduction in feeding is evident in fed or fasted animals, as well as in animal models of diabetes or obesity, and is probably associated with a reduced meal size (for review see Lutz and Meyer, 2015). Although it has a profound effect on the homeostatic aspects of feeding, the outcome on hedonic eating is more variable as amylin reduces the consumption of high-fat diet (Mietlicki-Baase et al., 2015) without altering the intake of peanut butter (Kalafateli et al., 2019b) or operant self-administration of a chocolate-flavored beverage (Kalafateli et al., 2019a). Throughout the body, amylin acts *via* its receptor, the amylin receptor (AMYR), which consists of one of two calcitonin receptors (CTRa and CTRb) and one of three receptor activity-modifying proteins (RAMP1-3). The CTRa shifts the selectivity of the AMYR toward amylin over calcitonin. The CTRa/RAMP1-3 protein complex is therefore thought to mediate the physiological properties of amylin. As amylin has a short half-life in blood, other amylinergic compounds are used in research. This includes salmon calcitonin (sCT), an agonist for both the AMYR and CTR (Christopoulos et al., 1999). However, its often referred to as an AMYR agonist as the ability of sCT to reduce feeding mimics that of amylin, and sCT exerts its anorexigenic properties *via* the AMYR (Lutz et al., 2000). To date, more selective AMYR agonists, including AM1213, and antagonists such as AC187, are available tools when investigating the interaction between amylinergic pathway and various behaviors.

Ghrelin is a 28-amino acid long peptide discovered to be the endogenous ligand to the growth hormone secretagogue receptor (GHSR-1A) (Kojima et al., 1999). Notably, GHSR-1A possesses a strong intrinsic activity and forms heterodimer complexes with other receptors in the brain (for review see Cornejo et al., 2021). Ghrelin is primarily produced in peripheral organs such as the stomach but may be locally produced in the brain (Lu et al., 2002; Cowley et al., 2003; Mondal et al., 2005). Ghrelin promotes food intake and weight gain and has historically been thought of as a hormone regulating hunger and various feeding-related behaviors, including the hedonic and homeostatic aspects (Tschop et al., 2000; Cornejo et al., 2021). A plausible relation between hunger, ghrelin and addiction was shown early as food restriction, known to enhance plasma ghrelin (Gualillo et al., 2002), enhances amphetamine and cocaine consumption in male rats (Carroll et al., 1979). Of further interest to addiction and reward, GHSR-1A can form complexes with dopamine receptors and modulate their activity in the mesocorticolimbic pathway and consequently regulate reward-related behaviors (Kern et al., 2012, 2015; Mustafa et al., 2021). Subsequently, mounting evidence during the past decade has shown that both ghrelin and the GHSR-1A play a substantial role in modulating addiction and addiction-related behaviors.

## Appetite-Regulatory Peptides in Substance Use Disorder

### GLP-1 in Addiction

#### Stage and Components of the Addiction Cycle and Sites of Action

**Figure 3:**
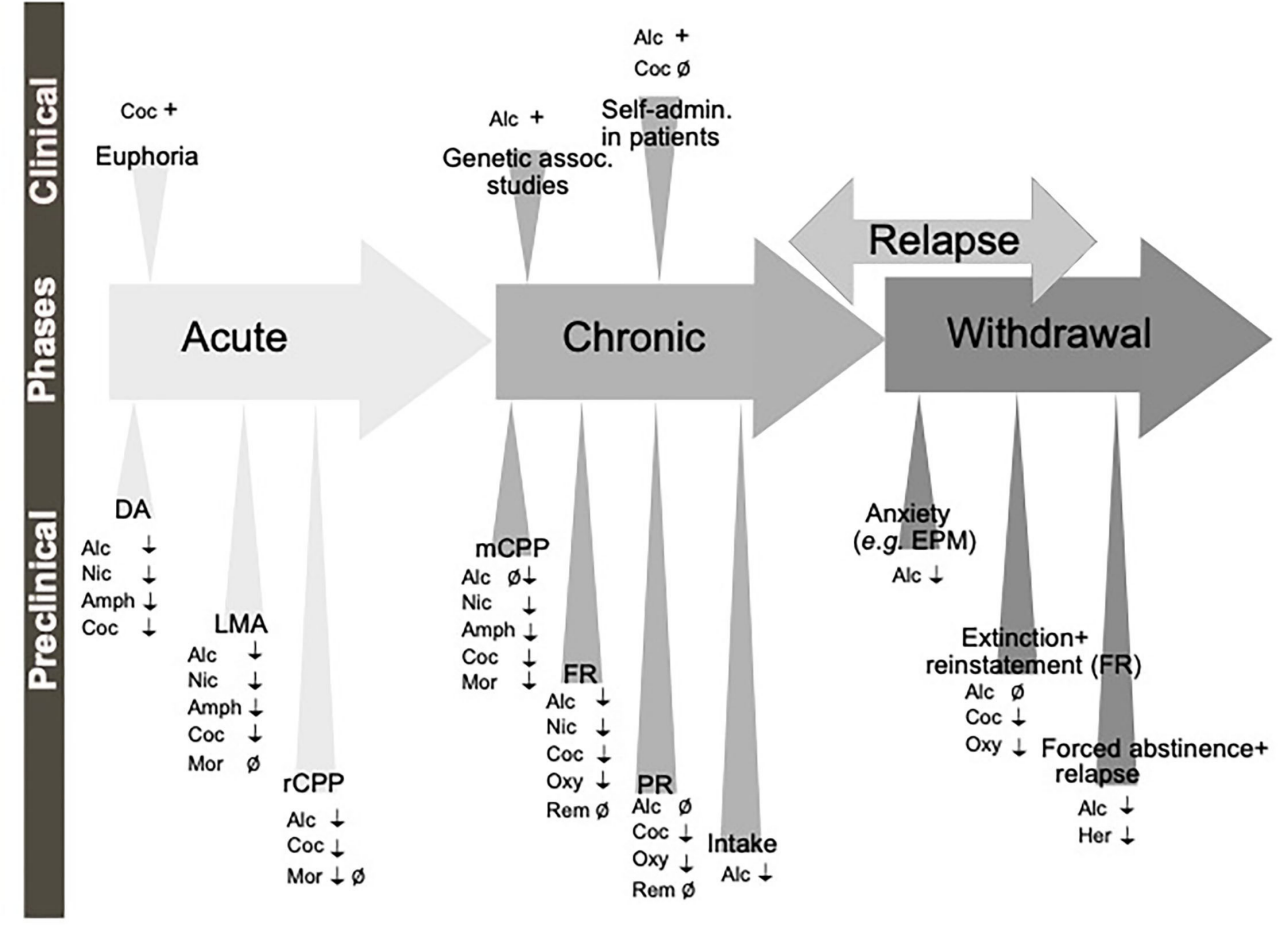
Schematic illustration of the different phases of the addiction cycle which GLP-1 signaling influence. GLP-1 or GLP-1 receptor agonists influence behaviors of the addiction cycle, related to alcohol (Alc), nicotine (Nic), amphetamine (Amph), cocaine (Coc), morphine (Mor), remifentanil (Rem), Oxycodone (Oxy), heroin (Her). Positive association (+), negative association (–), increases/enhances (↑), decreases/attenuates (↓), conflicting evidence or no effect (Ø) on drug effect. Dopamine release in nucleus accumbens (DA), locomotor stimulation (LMA), reward of conditioned place preference (rCPP), memory of alcohol reward in the CPP test (mCPP), motivation (FR, and PR), Alcohol and drug intake (Intake), functional magnetic resonance (fMRI).

**Figure 4:**
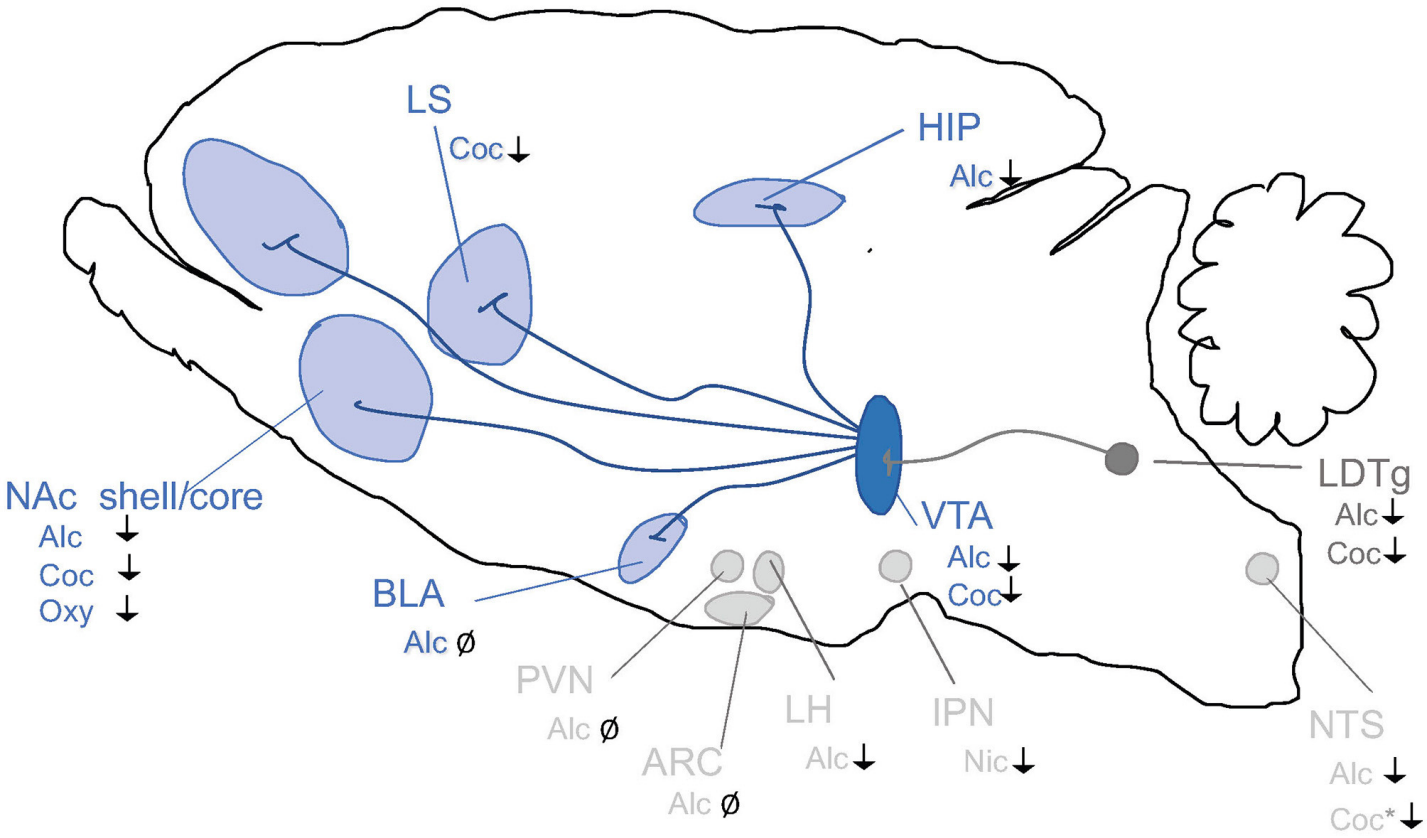
Schematic illustrations of brain regions important for the interaction between the GLP-1 pathway and addiction processes. GLP-1R agonists attenuate behaviors relating to reward and intake of alcohol (Alc), nicotine (Nic), cocaine (Coc) and oxycodone (Oxy). Nucleus tractus solitarii (NTS) of the brain stem, ventral tegmental area (VTA), nucleus accumbens (NAc) shell, laterodorsal tegmental area (LDTg), basolateral amygdala (BLA), lateral septum (LS) and hippocampus (HIP), paraventricular nucleus (PVN), arcuate nucleus (ARC) and lateral hypothalamus (LH) and interpeduncular nucleus (IPN). ↓ Shows an attenuation and Ø reflects no change. *Role shown indirectly.

In the non-addicted brain, the effect of modulating the GLP-1 system on alcohol-induced reward has been studied in rodent models of locomotor activation, conditioned place preference (CPP) and mesolimbic dopamine release (Figure 3), an interaction involving several reward-related areas of the brain (Figure 4). In male mice, the increase in locomotion following an injection of alcohol is decreased by systemic Ex-4 (Egecioglu et al., 2013c), as well as by Ex-4 injected specifically into NTS (Vallof et al., 2019b), LDTg, posterior VTA and NAc shell (Vallof et al., 2019a). Contrarily, injections of Ex-4 into anterior VTA does not affect alcohol-induced LMA (Vallof et al., 2019a). Of further interest are the findings that the systemically-mediated decrease of Ex4 is reversible by the antagonist Ex-9 into the NTS (Vallof et al., 2019b), suggesting that circulating GLP-1 exerts some if its effect on alcohol reward *via* NTS. A more direct way of measuring reward in rodents is the CPP test. Ex-4, GLP-1, and liraglutide injected systemically reverse the preference for the alcohol-conditioned chamber (Egecioglu et al., 2013c; Shirazi et al., 2013; Vallof et al., 2015). Finally, the neurochemical correlate of reward is also decreased by GLP-1R stimulation. Thus, Ex-4 and liraglutide both reverse the alcohol-induced increase in mesolimbic dopamine release in male mice (Egecioglu et al., 2013c; Vallof et al., 2015). Only one study has evaluated dopamine release following brain-region specific Ex-4 injections and found that indeed NTS-Ex-4 also reverses the ability of alcohol to increase accumbal dopamine (Vallof et al., 2019b).

Part of the process of transition from the acute non-addicted phase to chronic use is the consolidation of memories of reward gained from the drug that will drive behavior when opportunities for drug consumption arise in the future. In the memory CPP paradigm, systemic Ex-4 does decrease alcohol reward memory (Egecioglu et al., 2013c) however, liraglutide does not (Vallof et al., 2015). As seen above, the same studies found both to decrease reward when administered simultaneously with alcohol, suggesting that alcohol reward versus memory of alcohol reward is encoded by different brain regions, with different agonists targeting some and not others (Gabery et al., 2020; Salameh et al., 2020). A role for NTS (Vallof et al., 2019b) and NAc shell (Vallof et al., 2019a) in the dampening of reward conditioning memory by Ex-4 has been established, while posterior and anterior VTA, as well as LDTg, are not involved (Vallof et al., 2019a).

One human study includes data on the role of GLP-1 on motivation in non-dependent male and female drinkers. In this study, a polymorphism of the GLP-1R gene was associated with the rate of intravenous self-administration of alcohol and breath alcohol concentrations (Suchankova et al., 2015). In humans, some important features of addiction are an escalation of drug use, compulsive drug-taking, drug craving, inability to stop and frequent relapse after a period of abstaining. In rodent models, it is generally considered that an addiction phenotype, including neuroadaptations in the motivation, emotion and decision-making regions of the brain, occurs after multiple months of intermittent exposure, although some studies have also used shorter periods such as 4 weeks. GLP-1R agonists do decrease alcohol intake in models of chronic use. After a chronic (4-18 week) intermittent access two-bottle choice exposure rats treated with Ex-4, GLP-1, liraglutide, semaglutide acutely (Egecioglu et al., 2013c; Shirazi et al., 2013; Vallof et al., 2015; Marty et al., 2020) or with either liraglutide or dulaglutide repeatedly (Vallof et al., 2015, 2020) decrease alcohol intake and in some cases preference over water. Dulaglutide and liraglutide treatment continued to keep the alcohol intake at a level below controls even after discontinuation of treatment—for liraglutide, this lasted an additional two days (Vallof et al., 2015) while for dulaglutide it lasted for about 1 week (Vallof et al., 2020). The antagonist Ex-9 increased alcohol intake when injected peripherally after 4 weeks of intermittent exposure (Shirazi et al., 2013), whereas Ex-9 does not increase alcohol intake or reverse the alcohol intake decreased by liraglutide or semaglutide after 20-24 weeks of exposure (Marty et al., 2020). It is therefore plausible that endogenous GLP-1 controls alcohol intake in rodents exposed to alcohol for short but not long term. Besides single housed rats in the above studies, Ex-4 also decreases alcohol intake in chronically alcohol-exposed socially housed mice and vervet monkeys (Thomsen et al., 2017, 2019). In the case of vervet monkeys, the effect was replicated and even more pronounced with a liraglutide treatment (Thomsen et al., 2019).

Some anatomical specificity for the alcohol intake reduction following GLP-1R agonism has been elucidated. The importance of central GLP-1R has been shown using both a genetic model and pharmacologically. Ex-4 treatment reduces alcohol intake in control littermates, but not in mice with a neural GLP-1R knock-out (Sirohi et al., 2016). Three pharmacological approaches also converged on excluding a peripheral site of action: GPR119 agonists stimulate the production of GLP-1 in the gut, a DPP-4 inhibitor slows down its breakdown in the blood, and systemically injected Ex-9 primarily targets peripheral GLP-1R, neither of these manipulations affected alcohol intake in male rats (Marty et al., 2020). Within the brain, the region with the most solid data showing an effect of Ex-4 on alcohol intake is NAc shell—this has been shown by two studies in male (Vallof et al., 2019a; Colvin et al., 2020) and one study in female rats (Abtahi et al., 2018). NAc core was also shown to be involved in male rats (Colvin et al., 2020); however, in females, the same dose of Ex-4 failed to affect alcohol intake *via* NAc core (Abtahi et al., 2018). Furthermore, GLP-1R expression is higher in NAc shell in high-alcohol consuming than low alcohol-consuming rats (Vallof et al., 2019a). Although transgenic mice show that GLP-1R is predominantly expressed on neurons (Graham et al., 2020), fluorescently labeled Ex-4 has been shown to bind both neurons and astrocytes in the NAc shell, suggesting an intriguing glial-mediated mechanism of reward regulation. Evidence of a role of VTA in GLP-1 action on alcohol intake is more mixed. Despite using the same length of intermittent alcohol exposure and the same species, Colvin et al. (2020) reported VTA involvement in GLP-1 mediated alcohol intake decrease, while (Vallof et al., 2019a) found none. The latter study did, however, show the involvement of LDTg. In NTS, two doses of Ex-4 were tested, and the higher one did reduce alcohol intake in chronically alcohol-exposed rats (Vallof et al., 2019b). Additionally, Colvin and colleagues (Colvin et al., 2020) showed a role for dorsomedial hippocampus and lateral hypothalamus, but no involvement of basolateral amygdala, arcuate nucleus and paraventricular nucleus. Hippocampus also shows a trend for higher expression of the preproglucagon gene by high vs. low-alcohol consuming rats (Vallof et al., 2019a). In a human study, associations between polymorphisms in the GLP-1R gene was associated with AUD diagnosis (Suchankova et al., 2015). Animal models of self-administration generally reflect motivation for alcohol in the chronic use phase, as the animals need to be trained to self-administer alcohol for an extended period before their performance is stable enough to evaluate any pharmacological effects of GLP-1R agonists on this behavior.

Besides decreasing regular alcohol intake, GLP-1 modulates the motivation for alcohol as liraglutide decreases self-administration of alcohol in rats selectively bred to prefer alcohol (Vallof et al., 2015). To the best of our knowledge, only one recent publication defines a GLP-1R expressing brain area important for the motivation to consume alcohol. In this study, local infusion of Ex-4 into the habenula reduces the progressive ratio responding for alcohol in rats (Johnson et al., 2021).

When alcohol is no longer consumed for an extended period, humans as well rodents transition into a withdrawal state associated with specific abstinence symptoms. During withdrawal, rats show an elevated level of anxiety, which can be measured using the elevated plus maze paradigm. While acutely alcohol decreases anxiety measured by this test, chronic use followed by forced abstinence produces an increase in anxiety. Treatment with liraglutide (Sharma et al., 2014b) or a DPP-4 inhibitor, sitagliptin (Sharma et al., 2014a) both decreases the alcohol withdrawal-induced increase in anxiety. Another withdrawal-induced phenomenon that can be modeled in rodents is the post-abstinence escalation in drinking, also called relapse drinking. Ex-4 treatment in socially housed mice (Thomsen et al., 2017) and liraglutide treatment in rats (Vallof et al., 2015) are able to partially curb relapse drinking. On the other hand, using an oral self-administration paradigm, Dixon and colleagues (Dixon et al., 2020) showed no effect of Ex-4 on relapse alcohol self-administration after approximately five sessions of extinction, while Ex-4 did decrease baseline alcohol self-administration before extinction. Finally, repeated cycles of alcohol exposure followed by deprivation cause an even bigger increase in consumption during periods of availability. Using this paradigm, treatment with a GLP-1R agonist AC3174 was introduced during alcohol availability periods following five, six, and seven cycles of exposure-deprivation, showing a significant decrease during the 2nd week of treatment (Suchankova et al., 2015). The decrease persisted after the termination of GLP-1R agonist treatment.

Besides alcohol, GLP-1 signaling modulates the acute or chronic effects of various addictive drugs (Figure 3). In the acute period of pre-addiction, Ex-4 decreases locomotor activation induced by nicotine, amphetamine, cocaine but not morphine (Erreger et al., 2012; Egecioglu et al., 2013a,b; Sorensen et al., 2015; Bornebusch et al., 2019). Ex-4 additionally decreases nicotine-induced locomotor sensitization (Egecioglu et al., 2013a). Amphetamine-evoked hyperlocomotion has also been decreased by inhibiting DPP-4 (Lautar et al., 2005). Conversely, GLP-1R knockout mice show an enhanced locomotor stimulation by cocaine (Harasta et al., 2015) and this exaggerated response can be decreased back to wild-type levels by virally-mediated re-expression of GLP-1R in the lateral septum (LS) (Harasta et al., 2015). The LS contribution is somewhat specific to drug-induced hyperlocomotion; anxiety is increased by the full-body knockout but not rescued by LS overexpression (Harasta et al., 2015).

VTA is a region where fluoro-Ex4 binds to both neurons and astrocytes, and indeed GLP-1R agonists decrease the increased dopamine release from VTA to NAc induced by nicotine, amphetamine, or cocaine (Egecioglu et al., 2013a,b; Sorensen et al., 2015). Within the NAc, cocaine acts both on the core and shell subregions. The cocaine-induced increase in early immediate gene expression in NAc shell and striatum is decreased by Ex-4 (Sorensen et al., 2015), while in NAc core, Ex-4 suppressed the cocaine-induced phasic release of dopamine (Fortin and Roitman, 2017). Dopamine release induced by cocaine is also suppressed by Ex-4 in the lateral septum, and this effect is mediated by a decrease in dopamine transporter, which in turn is reduced *via* a mechanism involving 2-arachidonoylglycerol and arachidonic acid (Reddy et al., 2016). In the lateral septum, GLP-1R is expressed primarily by GABAergic neurons, and in GLP-1R KO mice, these particular neurons show reduced excitability (Harasta et al., 2015).

Importantly, increasing GLP-1R activation also reduces the reward of cocaine and morphine (Graham et al., 2013; Lupina et al., 2020) using DPP-4 inhibition and the memory of reward conditioned by nicotine, amphetamine, cocaine and morphine (Egecioglu et al., 2013a,b) both with Ex-4, (Lupina et al., 2020) with DPP-4 inhibition. In the case of cocaine, a neuroinflammatory mechanism has been shown to underlie the Ex-4 mediated reduction (Zhu et al., 2021). DPP-4 inhibition also decreases the reinstatement of conditioning following 5 sessions of morphine-free extinction. On the other hand, another study shows no effect of Ex-4 in reward conditioning by morphine in either wildtype or neuronal-specific GLP-1R knockout mice (Bornebusch et al., 2019). Transgenic models have confirmed these reward-reducing results of GLP-1 and narrowed down the effect to neuronal GLP-1R for amphetamine (Sirohi et al., 2016), lateral septum GLP-1R for cocaine (Harasta et al., 2015) and interpeduncular nucleus GLP-1R for nicotine (Tuesta et al., 2017).

As mentioned previously, studies of motivation for drugs in rodent models are usually performed in animals chronically exposed to the drug, since they have to be trained before they will reliably work for the drug. However, one study has performed a self-administration experiment in drug-naïve mice and found that Ex-4 decreases lever pressing for cocaine (Sorensen et al., 2015). Results from humans in a chronic use stage however show no decrease in the number of cocaine infusions self-administered or level of cocaine wanting following Ex-4 (Angarita et al., 2021). On the other hand, another study with cocaine-experienced human subjects found a positive correlation between GLP-1 levels in the blood and subjective high from cocaine, enhanced respiratory rate and elevated heart rate (Bouhlal et al., 2017). The same study found an overall decrease in GLP-1 after cocaine consumption (Bouhlal et al., 2017), a finding confirmed in the other cocaine human study (Angarita et al., 2021). However, in rats' GLP-1 increases after cocaine (You et al., 2019). Results from a different rodent model of drugs, specifically hallucinogenic drugs, show that the mescaline-induced scratching is reduced following a DPP-4 inhibitor (Lautar et al., 2005).

Just as in non-addicted brains, in chronic use rodent models, GLP-1R stimulation decreases responses to drugs. Self-administration models were used to show that Ex-4 or DPP-4 inhibition decreases responding for nicotine, while GLP-1R knockout increases it (Tuesta et al., 2017). Furthermore, nicotine increases the activation of GLP-1 producing neurons in NTS, and chemogenetic activation of this population decreases the response for nicotine (Tuesta et al., 2017). A neural circuit has been identified mediating this effect. Optogenetic stimulation of GLP-1 producing neurons in NTS stimulates interpeduncular nucleus (IPN) neurons while Ex-4 in IPN increases activity in cholinergic inputs from the medial habenula. Knocking down GLP-1R in the neural terminals from the medial habenula that terminate in IPN (which in turn receives GLP-1 from NTS) increases nicotine self-administration. Supportively, Ex-4 in IPN decreases nicotine responses while Ex-9 into this area increases them. One important downstream mechanism for this effect is cAMP, co-administering a non-active cAMP blocks the effect of Ex-4, while a non-active cGMP does not alter the Ex-4 evoked response (Tuesta et al., 2017). The effects of GLP-1 in modulating motivation for cocaine has been associated with VTA as Ex-4 infused into this brain region decreases motivation as measured by progressive ratio responding for cocaine but not sucrose, while knockdown of GLP-1R in VTA increases motivation for cocaine (Schmidt et al., 2016). In cocaine-experienced rats, Ex-4 enhanced intrinsic but not synaptic activity on medial spiny neurons in the NAc (Hernandez et al., 2019). The stress axis has been implied in this effect since cocaine increases the corticosterone levels, which in turn increased activation of GLP-1 producing neurons in NTS. Injecting corticosterone into the fourth ventricle, located close to NTS, decreases cocaine-motivated responding, while Ex-4 into VTA blocks this effect, suggesting that cocaine use activates NTS GLP-1-producing neurons projecting to the VTA through a corticosterone-mediated mechanism (Schmidt et al., 2016). A decrease in motivation for opioids has also been attributed to GLP-1. Oxycodone-experienced rats injected systemically with Ex-4 decrease their self-administration and progressive ratio responding for oxycodone, an effect that can be mimicked by Ex-4 injections specifically into NAc shell (Zhang et al., 2020). While this study used acute injections of Ex-4 in drug-experienced animals, another opioid study instead injected Ex-4 daily starting with drug-naïve mice and throughout the period of learning to self-administer remifentanil. In this context, Ex-4 had no effect on any parameters of responding for remifentanil in either wildtype or neural-knockout of GLP-1R mice (Bornebusch et al., 2019).

For the withdrawal stage of addiction, the effect of GLP-1 has been evaluated for cocaine, heroin and oxycodone. Extinction following cocaine self-administration was associated with decreased precursor preproglucagon mRNA expression in the NTS (Hernandez et al., 2018). In addition, injections of Ex-4 into VTA decreases cocaine-seeking that follows from extinction followed by a single priming dose of cocaine (Hernandez et al., 2018), while Ex-9 reverses this decrease to baseline levels. A similar effect was shown when relapse was triggered with drug-associated cues rather than a priming dose. Other studies report that the equivalent effect can be evoked with Ex-4 injected into NAc core or shell (Hernandez et al., 2019) or LDTg (Hernandez et al., 2020). The effect *via* LDTg has been identified as mediated *via* GABAergic neurons projecting to the VTA, as knocking down the GLP-1R in these neurons decreases the ability of Ex-4 to decrease cocaine-seeking after extinction followed by a priming dose of cocaine (Hernandez et al., 2020). Repeated daily Ex-4 injections during extinction in addition to Ex-4 on the day of reinstatement, decreased cue (lights above the levers), and heroin priming dose-induced heroin seeking after 16 days forced abstinence (Douton et al., 2021). After extinction from oxycodone and then drug-priming, reinstatement of the high level of responding for oxycodone seen in control animals is decreased by Ex-4 systemically or into NAc shell (Zhang et al., 2020).

#### Individual Differences

As we live in the era of personalized medicine, it has become important to understand whether particular characteristics of different individuals can predict how they will respond to treatments. This will allow to develop a better patient stratification strategy and tailor the treatment regimens accordingly. Unfortunately, there is so far limited literature on how sex/gender, age, genetic background, ancestral groups, medical co-morbidities or other factors influence the ability of GLP-1R agonists to aid individuals with alcohol or substance use disorder to reduce their drug intake and curb their addiction.

The majority of rodent studies have been performed on male subjects only, and most human studies do not analyze the results for men and women separately. The effect of dulaglutide on reducing alcohol intake shows that both sexes are responsive, although males show a stronger decrease and continue to decline their intake after treatment termination, which the females do not (Vallof et al., 2020). The effect of Ex-4 injected in NAc on decreasing alcohol intake has been shown in males by two studies (Vallof et al., 2019a; Colvin et al., 2020) and in females by one study (Abtahi et al., 2018). Overall, both sexes show alcohol intake decreases following Ex-4 injections in NAc shell, however only in males when Ex-4 is injected in NAc core instead (Colvin et al., 2020), although this discrepancy could also be explained by minor methodological differences between the two studies. Suchankova and colleagues (Suchankova et al., 2015) reported that certain *GLP1R* gene polymorphisms were associated with AUD, but only in men. The same study also reveals that individuals with specific *GLP1R* gene polymorphisms are more likely to suffer from AUD and show higher levels of intravenous alcohol self-administration. Although this study did report the age of participants, no interactions were reported between age and other factors in determining the effect of GLP-1R polymorphisms on alcohol-related outcomes. The relationships between GLP-1 and alcohol were also found to not differ by ancestral group, with both Caucasian and African-American individuals showing the same pattern (Suchankova et al., 2015). In a small pilot study, T2D patients treated with liraglutide decreased alcohol intake (Kalra, 2011); however, we do not know if this constitutes a different mechanism according to co-morbidity or illustrates a general pattern for humans in general, as there is no other published study showing the effect of a GLP-1R agonist on alcohol intake. Although the polymorphisms study, the wealth of data in rodents, one study in non-human primates and the ongoing clinical studies evaluating GLP-1R agonists on AUD are all indicative that GLP-1R agonists indeed would reduce alcohol intake in a majority of humans regardless of T2D status. An interesting study in rats that had undergone Roux-en-Y gastric bypass (RYGB) or sham surgery shows that Ex-4 decreases alcohol intake in sham but not RYGB rats (Davis et al., 2012). The latter had strongly increased their circulating levels of GLP-1 and had also decreased their alcohol intake compared to sham controls, as well as their own pre-surgery baselines. CPP for the alcohol-paired chamber was also decreased by RYGB surgery, indirectly suggesting that the increased GLP-1 action in these rats might be responsible for the decreased alcohol reward (Davis et al., 2012).

#### Future Perspectives

As mentioned, multiple GLP-1R agonists are used in the clinic, and many of them have also been used in the above research on addiction. Comparing how different or similar they act on various phases and components of addiction is helpful not only to understand their clinical usefulness and limitations, but also to give us clues to the mechanism of GLP-1 action on addiction processes. Since the agonists have differential rates of blood brain barrier penetration and thus different distribution of effects in the brain, we can combine this knowledge with what we know about brain-region specific effects to understand better the neural circuits and mechanisms underlying the effect. Downstream targets of GLP-1R involved in drug addiction reduction could be investigated as candidates for future treatments, possibly more specific to the treatment of a particular addiction. Additionally, two recent developments that could present interesting avenues for research in addiction are the new modality positive allosteric modulators of GLP-1R and co-agonists of GLP-1 and glucose-dependent insulinotropic polypeptide.

Possible limitations to using GLP-1R agonists for treating SUD are nausea, taste aversion and excessive body weight loss. On the other hand, in addition to the brain effects described above, GLP-1R agonists could also act on intestinal hyperpermeability and gut microbiota diversity, probably in a way that protects against the harmful effects of chronic drug use. Dulaglutide might thus be of extra interest as a clinical study with dulaglutide reporting a low number of aversive effects (Winzeler et al., 2021). Finally, GLP-1 also increases anti-inflammatory cytokines, providing another beneficial effect relevant for certain types of addictions.

GLP-1 signaling is implicated in many socio-cognitive-emotional behaviors, and it is important to consider how this regulation will interact with the ability of GLP-1R agonists to treat addiction. GLP-1R activation acts on the stress axis acutely by increasing physiological stress and anxiety-related behavior in rodents. On the other hand, chronic treatments with GLP-1R agonists or genetic knockdown studies tend to show an decrease in the same stress-related parameters, although results from human studies are not as clear (reviewed by Guerrero-Hreins et al., 2021). As we have seen, corticosterone increases activation of GLP-1 producing neurons (Schmidt et al., 2016) but acute stress also decreases preproglucagon gene expression in NTS and GLP-1 availability in the paraventricular nucleus (Zhang et al., 2009). NTS GLP-1 neurons are activated in response to a large unexpected meal but not by ad libitum homeostatic feeding—this evidence has been used to propose that NTS GLP-1 neurons are primarily responsive to stress. Since NTS GLP-1 neurons also express high levels of GLP-1R and, furthermore, are probably accessible to compounds that do not pass the blood brain barrier, treating AUD/SUD patients with GLP-1R agonists will probably also affect their NTS activation. Therefore, it could be relevant to consider the interaction of stress, including chronic intermittent stress and chronic substance use, in terms of activation of NTS GLP-1 neurons. Stress is a well-known factor that predisposes to the development of addiction; therefore, it is important to test the effects of GLP-1R agonists on rodent models of addiction under conditions of chronic stress.

Acutely GLP-1R agonists have no effect on depression-like behavior, but chronically, GLP-1R agonists decrease depression-like behavior (reviewed by Guerrero-Hreins et al., 2021). One study found that GLP-1 increased depressive-like behavior; however, the subjects were diabetic and in a fasting state, suggesting that the anti-depressive effect of GLP-1 could be dependent on metabolic status (Palleria et al., 2017). Overall, the promising anti-depressive effects of GLP-1 could partially explain the positive results seen in treating addiction-like behavior too, and future studies should investigate how much the anti-depressive effect contributes to decreasing substance use in rodent models as well as humans.

Increasing evidence is pointing toward an effect of GLP-1 on improving memory and cognition, and GLP-1R agonists are being investigated for the treatment of neurodegenerative disorders. Some of the neuroprotective effects are mediated by increasing neurogenesis and synaptic plasticity (reviewed by Kim et al., 2020). This opens an intriguing possibility that one beneficial effect that GLP-1R agonists could play in treating addiction is to open new windows of learning through neuroplasticity. An important component of addiction is habit formation. Thus, giving the brain a chance to form new different connections during periods of abstinence could aid in breaking down the learned associations between external and internal cues and substance use.

### Amylin in Addiction

#### Stage and Components of the Addiction Cycle and Sites of Action

**Figure 5:**
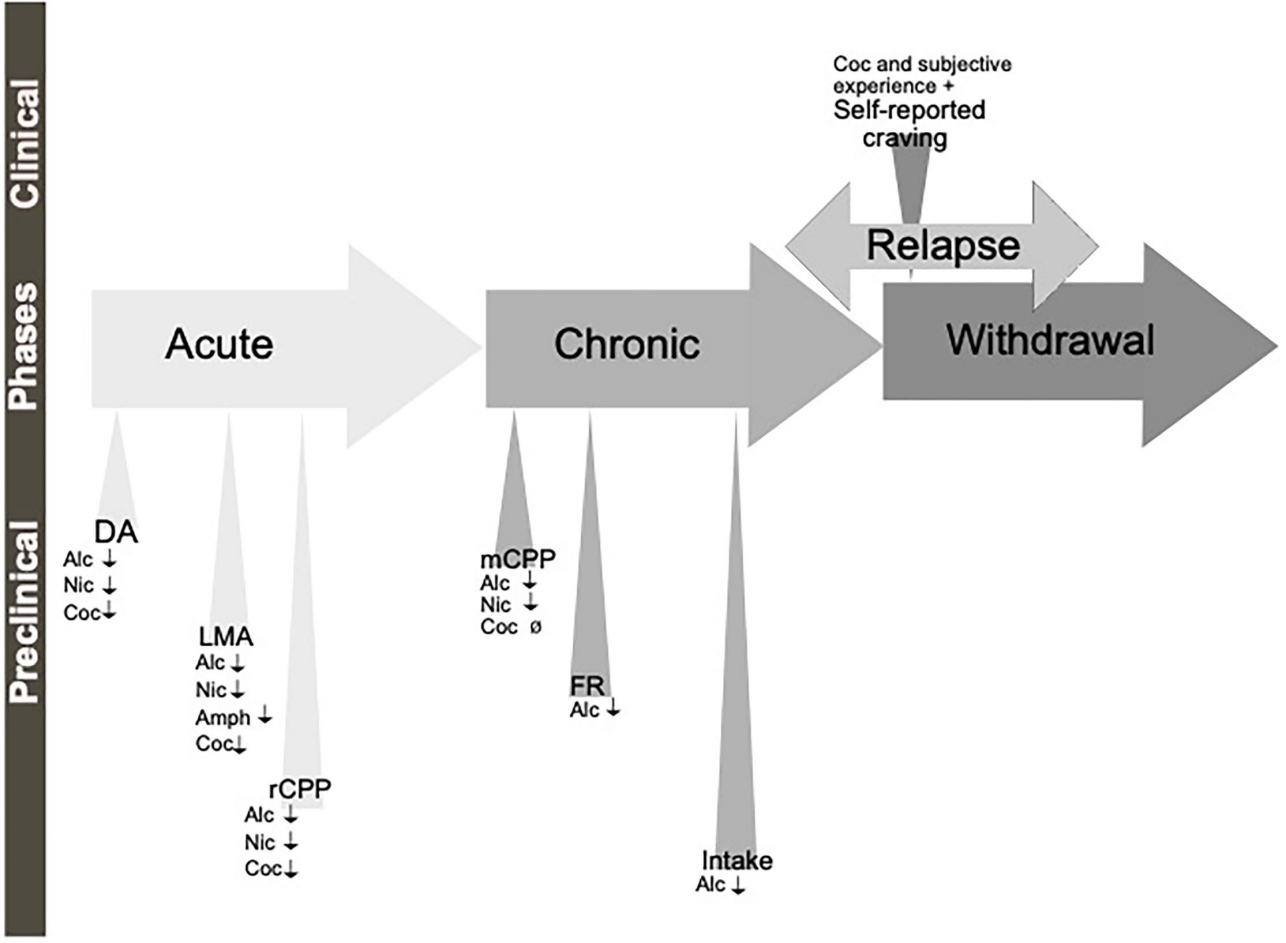
Schematic illustration of the different phases of the addiction cycle which amylin signaling influence. Amylin or amylin receptor agonists influence behaviors of the addiction cycle, related to alcohol (Alc), nicotine (Nic), Amphetamine (Amph) or cocaine (Coc). Positive association (+), decreases/attenuates (↓), conflicting evidence or no effect (Ø) on drug effect. Dopamine release in nucleus accumbens (DA), locomotor stimulation (LMA), reward of conditioned place preference (rCPP), memory of alcohol reward in the CPP test (mCPP), motivation (FR, and PR), Alcohol and drug intake (Intake), functional magnetic resonance (fMRI).

**Figure 6:**
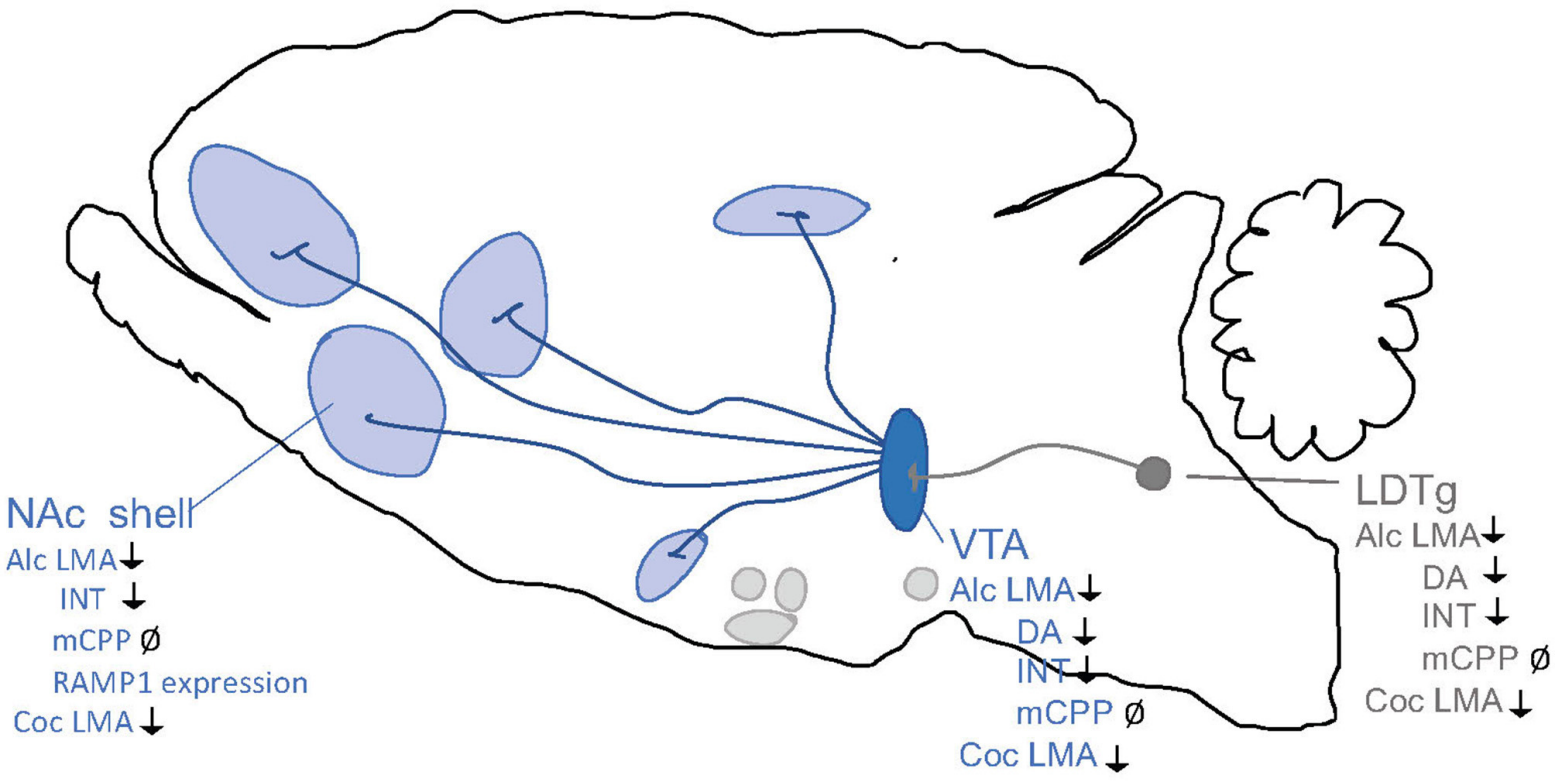
Schematic illustrations of brain regions important for the interaction between the amylin pathway and addiction processes. Salmon calcitonin (sCT) attenuates behaviors of alcohol (Alc) and cocaine (Coc) like locomotor stimulation (LMA), conditioned place preference (CPP), dopamine release in NAc shell (DA), and alcohol intake (INT) *via* areas above. Ventral tegmental area (VTA), nucleus accumbens (NAc) shell, laterodorsal tegmental area (LDTg). ↓ Shows an attenuation and Ø reflects no change.

Although less studied than GLP-1, activation of the amylinergic pathway regulates both the acute and chronic phases of alcohol-related behaviors in the addiction cycle (Figure 5). Initial studies of male mice show that a systemic administration of sCT regulates the acute intoxication phase, which is associated with alcohol's rewarding properties. Specifically, sCT prevents the ability of alcohol to cause a hypermotion, dopamine release in NAc shell and alcohol reward in the CPP test (Kalafateli et al., 2019b). Similarly, repeated sCT pre-treatment attenuates the locomotor stimulation associated with alcohol even when sCT is no longer in the body (Kalafateli et al., 2020). For this interaction, central mechanisms have been implied as immunohistological studies show that systemic administration of sCT penetrates the brain and binds to areas important for reward processing, such as the LDTg, VTA or NAc shell (Zakariassen et al., 2020; Kalafateli et al., 2021b). Supportively, sCT infused locally into the aforementioned areas attenuates the accumbal dopamine enhancement and locomotor stimulation associated with alcohol areas (Kalafateli et al., 2021b) (Figure 6). In chronic models of alcohol use disorder in male rats, a reduced alcohol intake is observed following systemic administration of sCT, either given acutely (Kalafateli et al., 2019b) or repeatedly (Kalafateli et al., 2019a). These initial studies cannot selectively dissociate the importance of AMYR compared to CTR as sCT activates both receptors. However, the findings that an acute systemic administration of the AMYR antagonist (AC187) increases the alcohol intake in male rats (Kalafateli et al., 2019a) and as selective AMYR agonist (AM1213) reduces alcohol drinking in male and female rats (Kalafateli et al., 2021c), supports the higher importance of AMYR than CTR for alcohol consummatory behaviors. In this alcohol drinking model in male rats, local infusion of sCT into the LDTg, VTA, or NAc shell reduces alcohol drinking (Kalafateli et al., 2021b) (Figure 6). On that note, rats consuming high amounts of alcohol display lower expression of the RAMP1 gene in the NAc shell (Kalafateli et al., 2019a) compared to rats consuming low amounts of alcohol. As the chronic phase of AUD involves relapse drinking, the findings that sCT prevents the escalated alcohol intake following abstinence in male rats (Kalafateli et al., 2019a) are of interest. Besides, in male alcohol-preferring rats who self-administer alcohol in the operant model, sCT reduces the motivation to consume alcohol (Kalafateli et al., 2019a). Together these findings display that the amylinergic pathway modulates both the acute intoxicating and the chronic phase of the AUD cycle, whereas studies on the withdrawal phase are missing. The findings that sCT, when infused systemically (Kalafateli et al., 2019b) but not into reward-related areas (Kalafateli et al., 2021b), blocks the memory of alcohol reward in the CPP test, indicating that the amylinergic pathway might prevent AUD processes both through reducing the euphoric properties of alcohol and by reducing the memory thereof.

Besides alcohol, there is evidence implying that amylin signaling modulates the behavioral responses to other addictive drugs (Figure 5). Thus, the ability of amphetamine (Twery et al., 1986; Clementi et al., 1996), cocaine (Kalafateli et al., 2021a) or nicotine (Aranas et al., 2021) to cause a hypermotion in male rodents is reduced by systemic administration of either amylin or sCT. The locomotor stimulatory properties of cocaine involve activation of the amylinergic pathway in the LDTg, VTA or NAc shell (Kalafateli et al., 2021a) (Figure 6). Similarly, systemic administration of sCT declines the enhanced dopamine levels in NAc shell induced by cocaine (Kalafateli et al., 2021a) or by nicotine (Aranas et al., 2021). Whereas systemic administration of sCT prevents the memory of nicotine reward (Aranas et al., 2021) in the CPP test, it does not influence this behavior induced by cocaine (Kalafateli et al., 2021a). Although human association studies between drugs of abuse and amylin are limited, one study revealed that the serum concentrations of amylin are associated with the subjective responses (e.g., anxiousness and increased heart rate) following an intravenous infusion of cocaine (Bouhlal et al., 2017).

#### Individual Differences

While these preclinical studies predominantly are conducted in male rodents, only one study revealed that a selective AMYR agonist reduces alcohol intake in male and female rats (Kalafateli et al., 2021c). It should however be noted that there are some differences in response between genders, as the initial reduction in alcohol drinking returns to baseline consumption in females, whereas the alcohol intake during the later session is elevated in males (Kalafateli et al., 2021c).

#### Future Perspectives

For the future, both additional preclinical and clinical studies are needed. As there only is one study on female rats, additional studies including both genders are necessary as possible gender similarities and differences must be identified. So far, only one study has used a more selective AMYR agonist; therefore, additional studies separating the role of AMYR versus CTR are important. Furthermore, the plausible role of endogenous amylin for addictive behaviors should be evaluated in experiments using an AMYR antagonist. The exact mechanisms, including the neurocircuits, brain regions and second messenger systems, mediating the interaction between AMYR and addictive drugs remains to be determined in detail. To date, only a few studies have addressed this, and as summarized in Figure 6, the acute and chronic phases of alcohol- (Kalafateli et al., 2021b) or cocaine- (Kalafateli et al., 2021a) related behaviors are mediated *via* AMYR within the LDTg, VTA and NAc shell, and possibly involving dopamine and serotonin signaling within the VTA (Kalafateli et al., 2020) and other reward-related areas (Kalafateli et al., 2021c). Whereas central mechanisms have been suggested, stress responses and altered metabolism of alcohol have been excluded (Kalafateli et al., 2019b). As the mechanisms modulating this interaction most likely are complex, future molecular and neurochemical studies should address this knowledge gap. Other appetite-regulatory peptides like GLP-1 attenuate the abstinence phase of alcohol; such studies are warranted for amylin.

Besides these additional preclinical studies, human studies are needed as only one plasma-association study reveal some interaction between circulating amylin and cocaine experience (Bouhlal et al., 2017). First, a plausible association between the plasma levels of amylin and the different phases of the addiction cycle, like intoxication, craving, abstinence, and relapse, should be explored, as they have been done for ghrelin. Register studies could tentatively explore the alcohol/drug use in diabetic patients treated with the AMYR agonist, pramlintide, compared to those with other medications. In the long run, the possibility that pramlintide or other more selective AMYR agonists, which currently are being investigated for the treatment of obesity, should be explored as possible anti-AUD cessations. Such studies could be either conducted as an RCT or as human laboratory studies. It should also be considered that AMYR activation might enhance the cognitive deficits observed in some AUD patients, as activation of this pathway increases cognition and memory function (Grizzanti et al., 2018). Another subtype of patients who might benefit from treatment with these amylin compounds is AUD patients with obesity as these compounds also decrease weight (for review see Lutz and Meyer, 2015). Worth mentioning, activation of AMYR increases the latency and decreases the frequency of ejaculation of male rats in a sexual interaction paradigm (Clementi et al., 1999), indicating that AMYR might modulate behavioral addictions. Future studies exploring this possibility should be conducted.

### Ghrelin in Addiction

#### Stages and Components of the Addiction Cycle and Sites of Action

**Figure 7:**
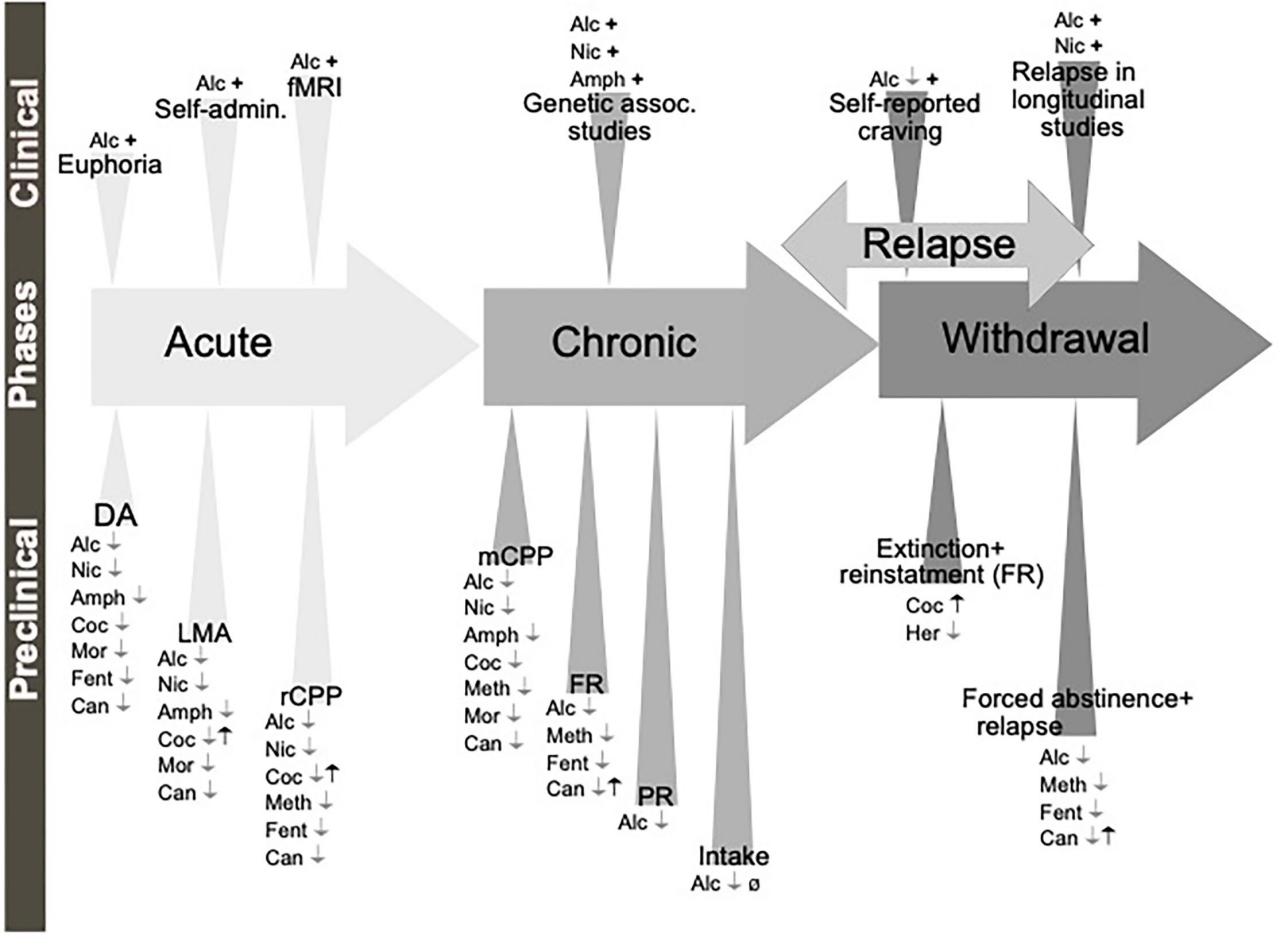
Schematic illustration of the different phases of the addiction cycle which ghrelin and GHSR-1A influence. Ghrelin (black symbols) and GHSR-1A receptor antagonists or reverse agonists (gray symbols) influence behaviors of the addiction cycle, related to alcohol (Alc), nicotine (Nic), amphetamine (Amph), cocaine (Coc), methamphetamine (Meth), morphine (Mor), fentanyl (Fent), heroin (Her) and Cannabinoids (Can). Dopamine release in nucleus accumbens (DA), locomotor stimulation (LMA), reward of conditioned place preference (rCPP), memory of reward in the CPP test (mCPP), motivation in fixed ratio (FR) or progressive ratio (PR) protocols, alcohol or drug intake (Intake), functional magnetic resonance (fMRI). Positive association (+), increases/enhances (↑), decreases/attenuates (↓), conflicting evidence or no effect (Ø) on drug effect.

Ghrelin substantially modulates the acute rewarding effects associated with drugs of abuse (Figure 7), and GHSR-1A is expressed in key regions of reward circuitry in both rodents (Guan et al., 1997; Zigman et al., 2006; Landgren et al., 2011) and humans (Deschaine et al., 2021). Thus, direct application of ghrelin increases the activity of VTA dopamine cells in both rat and mouse brain slices, an effect mediated by the GHSR-1A (Abizaid et al., 2006). Additionally, ghrelin promotes dopamine release in NAc (Jerlhag et al., 2006), as well as enhances dopamine release caused by nicotine when applied to striatal rat brain slices (Palotai et al., 2013). This effect could be reversed by applying the GHSR-1A antagonist GHRP-6. These results are complemented by an abundance of *in vivo* studies showing that ghrelin administered either peripherally or centrally, into the intracerebroventricular space (i.c.v.), induces the dopaminergic reward pathway and dopamine release in NAc in rodents (Abizaid et al., 2006; Jerlhag, 2008; Quarta et al., 2009; Jerlhag et al., 2011a; Edvardsson et al., 2021). Furthermore, ghrelin appears to act on several levels of this pathway, as local injections in the VTA or LDTg independently stimulates dopamine release in NAc (Jerlhag et al., 2007). The biochemical and electrophysiological observations that ghrelin stimulates the mesolimbic dopamine pathway is supported by substantial behavioral data. Thus, acute systemic or central administration of ghrelin has been shown to increase locomotor activity (Jerlhag et al., 2006, 2011a; Jensen et al., 2016; Suchankova et al., 2016a; Cornejo et al., 2018) and to induce reward in the CPP test (Jerlhag, 2008).

Due to its effects on the reward circuitry, ghrelin and the GHSR-1A appear to play essential parts in the rewarding effects of alcohol and drugs of abuse. As such, peripheral treatment of JMV2959, i.c.v. administration of the GHSR-1A antagonist BIM28163, or genetic suppression utilizing GHSR-1A knockout mice, attenuates the acute phase of alcohol as measured by locomotor stimulation, the reward of alcohol in the CPP test and dopamine overflow in NAc (Jerlhag et al., 2009; Suchankova et al., 2016a; Edvardsson et al., 2021). Interestingly, in GHSR knockout mice, alcohol appears to be unable to induce the above-mentioned behaviors (Jerlhag et al., 2011b; Bahi et al., 2013), suggesting that the GHSR-1A might not only play a key role but may even be required for alcohol to elicit its rewarding properties. This is supported by human studies as higher plasma ghrelin levels have been associated with a more subjective intensity and longer response to alcohol in social drinkers (Ralevski et al., 2017).

**Figure 8:**
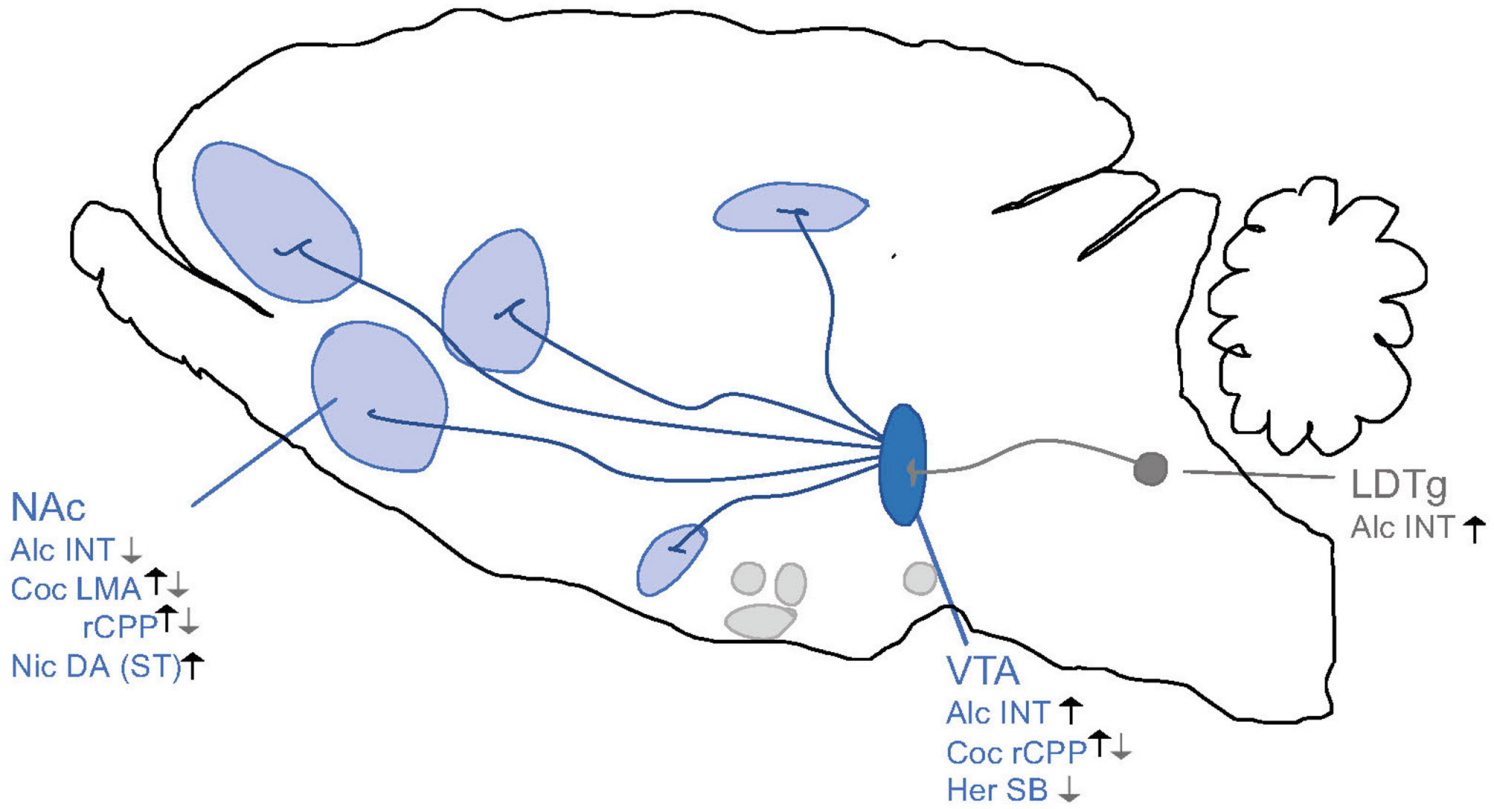
Schematic illustrations of brain regions important for the interaction between the ghrelin pathway and addiction processes. Ghrelin enhances (black arrows) whereas GHSR-1A antagonists (gray arrows) attenuates behaviors of alcohol (Alc), nicotine (Nic), cocaine (Coc) and heroin (Her) like locomotor stimulation (LMA), reward of conditioned place preference (rCPP), dopamine release in NAc shell (DA), alcohol or drug intake (INT) and seeking behaviors (SB) *via* areas above. Ventral tegmental area (VTA), nucleus accumbens (NAc), striatum (ST), laterodorsal tegmental area (LDTg). Increases/Enhances (↑), decreases/attenuates (↓), conflicting evidence or no effect (∅) on drug effect.

As antagonists, such as JMV2959 or BIM28163, attenuate these acute alcohol effects, the GHSR-1A emerges as an attractive drug candidate for AUD treatment. However, it is important to distinguish between acute rewarding effects and the long-term effects of alcohol, i.e., addiction and the development thereof. It is therefore of interest that the effect of ghrelin on alcohol consumption is evident even after long periods of consumption (Figure 7). For instance, ghrelin administered i.c.v., or bilaterally into VTA or LDTg enhances alcohol consumption in mice that previously consumed alcohol for 9 weeks (Jerlhag et al., 2009) (Figure 8). In the same study, peripheral administration of JMV2959 reduces alcohol consumption; a finding replicated in a similar study using high-consuming male rats, showing that JMV2959 reduces both alcohol consumption and preference to alcohol (Landgren et al., 2012). Accordingly, systemic administration of the GHSR-1A antagonists JMV2959 or [DLys3]-GHRP-6 decreases alcohol intake in male mice (Bahi et al., 2013; Gomez et al., 2015), male rats (Kaur and Ryabinin, 2010; Gomez and Ryabinin, 2014; Gomez et al., 2015) and male prairie voles (Stevenson et al., 2015), which consumed alcohol for shorter periods of time prior to testing. Additionally, both acute and repeated treatment with JMV2959 reduces voluntary alcohol intake in rats consuming alcohol for two, five and eight months (Suchankova et al., 2013b). Intriguingly, in this study, the treatment effect appeared to have a more marked effect in rats consuming for more extended periods of time, and there did not appear to occur any tolerance for the treatment or rebound increase in alcohol intake following discontinuation. Moreover, expression of GHSR in the VTA was negatively correlated with alcohol consumption with no effect in other brain areas. However, alcohol's effect on the expression of GHSR in the VTA has been subject to debate as a similar study found that GHSR expression was elevated in several other reward areas (Landgren et al., 2011), while a recent post-mortem study showed that central expression of ghrelin-related genes showed no difference between controls and AUD patients (Deschaine et al., 2021). In humans, polymorphisms in the gene encoding for GHSR have been associated with high alcohol intake in a Spanish cohort (Landgren et al., 2008), higher AUD identity score in a Finnish cohort (Suchankova et al., 2016b), and AUD type 2 (i.e., early-onset and heritability onset) in a Swedish female cohort (Landgren et al., 2010).

On a related note, it has been observed that chronic alcohol exposure reduces the levels of circulating ghrelin (Szulc et al., 2013; Yoshimoto et al., 2017) in rodents, whereas another study could not replicate this finding (Landgren et al., 2012). Either way, the relevance of circulating ghrelin in AUD is controversial, as the passage of circulating ghrelin to the brain in general might be limited (Grouselle et al., 2008; Sakata et al., 2009; Furness et al., 2011; Pirnik et al., 2011; Schaeffer et al., 2013). Thus, one study found that systemic administration of ghrelin increases alcohol intake in rats (Cepko et al., 2014). Contrarily, another study found no effect of peripheral ghrelin on alcohol intake (Lyons et al., 2008) and neutralizing circulating ghrelin using the oligonucleotide NOX-B11-2 does not appear to alter alcohol intake or alcohol reward in rodents (Jerlhag et al., 2013). Therefore, no major conclusions should be drawn from these data, and future studies will have to determine the effect circulating ghrelin levels and expression of alcohol-related behaviors.

An important part of chronic alcohol or drug use is the motivation to consume, which can be studied using an operant self-administration procedure. Acute systemic administration of a GHSR-1A antagonist (JMV2959 or [DLys3]-GHRP-6) reduces the operant self-administration of alcohol in male rats (Landgren et al., 2012; Gomez et al., 2015). A finding also obtained after central infusion of JMV2959 in NAc female rats (Abtahi et al., 2018). Accordingly, GHSR knockout rats self-administer less alcohol than wild-type (Zallar et al., 2019). These findings appear to translate into humans as an inverse GHSR-1A agonist, PF-5190457, decreases self-reported cue-elicited craving in AUD patients in a small clinical study (Lee et al., 2020b). Moreover, JMV2959 prevents the memory of alcohol reward in the CPP test (Jerlhag et al., 2009). Arguably, the most critical state of AUD and SUD treatment is during withdrawal, where relapse into alcohol drinking or drug use is a common phenomenon. An important factor for relapse is craving for alcohol or drugs, particularly cue-elicited cravings, which might be a formidable challenge even after long periods of being sober. The mesolimbic dopamine reward circuit has been implicated not only for alcohol reward in general, but specifically for alcohol craving and relapse vulnerability (Heinz et al., 2009; Koopmann et al., 2019). Interestingly, relapsing rats in this model show increased dopamine content in NAc and medial prefrontal cortex (Hadar et al., 2017), further strengthening the role of the dopamine reward circuit during relapse. Since ghrelin has been shown to affect this system substantially, GHSR-1A appears as an appealing target for treating craving and relapse drinking in AUD. Indeed, JMV2959 decreases relapse drinking in alcohol deprived rats (Suchankova et al., 2013b). In fact, alcohol intake was in this study reduced to the same level as the baseline, i.e., before they were re-introduced to alcohol. In humans, an abundance of studies has shown an association between craving and plasma ghrelin. However, such plasma-association studies should be considered carefully, as the role of circulating ghrelin in AUD is unclear. Nevertheless, plasma ghrelin has been positively associated with higher alcohol craving in abstaining AUD patients (Addolorato et al., 2006; Wurst et al., 2007; Leggio et al., 2012). Taking into account that ghrelin can be in both acylated, traditionally called the active, and des-acylated forms, more recent studies have shown that acylated, rather than total, plasma ghrelin was associated with alcohol craving (Koopmann et al., 2012, 2019; Sha et al., 2021). Furthermore, functional magnetic resonance imaging studies have demonstrated a positive correlation between active plasma ghrelin and neuronal alcohol-cue reactivity (Bach et al., 2019; Koopmann et al., 2019). Strikingly, the GHSR-1A inverse agonist, PF-5190457, declines self-reported alcohol cue-elicited craving in a bar-like environment in AUD patients in early withdrawal (Lee et al., 2020b).

In comparison to alcohol, the association between the use of other addictive substances and ghrelin is less explored. Nevertheless, ghrelin and GHSR are key components in the rewarding effects of other addictive drugs (Figure 7). For example, ghrelin enhances dopamine release in NAc caused by nicotine (Palotai et al., 2013). In contrast, the antagonist JMV2959 attenuates nicotine-induced dopamine release in NAc, locomotor sensitization and stimulation as well as attenuates reward in the CPP test in rodents (Jerlhag and Engel, 2011; Wellman et al., 2011, 2013; Palotai et al., 2013). Similarly, systemic or central administration of ghrelin potentiates the effects of central stimulants (e.g., amphetamine and cocaine) on locomotor stimulation and CPP (Wellman et al., 2005; Davis et al., 2007; Jang et al., 2013; Schuette et al., 2013; Dunn et al., 2019), whereas GHSR-1A antagonists attenuate these behavioral responses in rodents (Jerlhag et al., 2010; Abizaid et al., 2011; Clifford et al., 2012; Suchankova et al., 2016a; Havlickova et al., 2018; Wenthur et al., 2019; Edvardsson et al., 2021). Further, the acute rewarding effect of opiates and its relationship with the GHSR-1A is evident as JMV2959 inhibits the dopaminergic release in NAc and CPP caused by morphine or fentanyl (Engel et al., 2015; Sustkova-Fiserova et al., 2016, 2020; Jerabek et al., 2017). Besides, JMV2959 reduces morphine-induced locomotor stimulation and stereotypic behavior in male rodents (Sustkova-Fiserova et al., 2014). Additionally, JMV2959 have been shown to inhibit elevated accumbal dopamine levels and CPP caused by cannabinoids in male rats (Charalambous et al., 2020, 2021). Regarding chronic use and SUD, plasma ghrelin has been associated with cocaine-seeking and expectancy (Tessari et al., 2007; You et al., 2019), whereas systemic administration of JMV2959 reduces self-administration of methamphetamine in rats (Havlickova et al., 2018). Accordingly, JMV2959 reduces intake of fentanyl and fentanyl-seeking behavior in the operant self-administration model in male rats (Sustkova-Fiserova et al., 2020). Furthermore, human genetic studies have determined an association between genetic variation within the GHSR-1A gene and smoking (Landgren et al., 2010; Suchankova et al., 2016b), as well as amphetamine dependence (Suchankova et al., 2013a).

During withdrawal, higher plasma ghrelin appears to increase the risk of relapse during smoking caseation in humans (Al'absi et al., 2014). On the other hand, JMV2959 prevents drug-seeking and relapse behavior to methamphetamine in rats (Havlickova et al., 2018). Similarly, JMV2959 administered peripherally or centrally into the VTA reduces drug-seeking and relapse behavior for opiates in rodents (D'cunha et al., 2020; Sustkova-Fiserova et al., 2020). Further, ghrelin increases the self-administration and seeking behavior for cannabinoids, whereas JMV2959 decreases these behaviors (Charalambous et al., 2021).

#### Individual Differences

One of the most obvious but relatively unaddressed issues is that of sex differences. For instance, phase 1a/b clinical trials of the inverse GHSR-1A agonist PF-5190457 have recently been reported (Denney et al., 2017; Lee et al., 2020a,b). Although the drug appears promising, only one woman was included in these trials, albeit only a small study population was included in these reports. The male bias is also evident in preclinical research, which may become a translational problem in the future. As reviewed elsewhere (see Becker et al., 2017), sex differences have been established on several components of the addiction cycle and have also been observed for ghrelin in AUD patients (Wurst et al., 2007). Thus, preclinical and clinical research of the ghrelin system and its contribution to addiction disorders must account for possible sex differences.

Another interesting phenomenon is that gastric bypass surgery increases the risk of developing AUD (King et al., 2017). This effect has been partially attributed to changes in gut hormones, including ghrelin, following gastric surgery (for review see Blackburn et al., 2017; Orellana et al., 2019). Thus, obese rats that have undergone a vertical sleeve gastrectomy or RYGB show an increased alcohol intake, an effect reversed by the GHSR-1A antagonists JMV2959 or [DLys3]-GHRP-6 (Hajnal et al., 2012; Orellana et al., 2018, 2021). However, the extent to which gastric surgery affect plasma ghrelin levels is uncertain as current literature reports inconsistent results (Chambers et al., 2013; Orellana et al., 2019). Another hypothesis is that chronically reduced levels circulating of ghrelin caused by obesity (Tschop et al., 2001) lead to increased sensitivity of GHSR-1A, and consequently, D2-receptors (Dunn et al., 2012). This is supported by experimental observations that vertical sleeve gastrectomy or RYGB rats are more sensitive to the reduction of alcohol intake by GHSR-1A antagonists. However, it is important to consider that many of the patients undergoing gastric bypass surgeries display other risk factors for developing AUD or SUD. For instance, binge- and over-consumption behaviors as well as psychiatric comorbidities such as depression (Legenbauer et al., 2009).

#### Future Perspectives

The above-mentioned data provide strong evidence for ghrelin systems association to addiction, and in particular to AUD. Although the evidence for other addictive substances is not as extensively explored, further studies are warranted to fully establish the relationship between the ghrelin system and SUD.

It appears evident that ghrelin and GHSR-1A are deeply integrated into the mesolimbic dopaminergic reward pathway. GHSR-1A is expressed in several reward-related brain areas, including VTA and LDTg (Guan et al., 1997; Zigman et al., 2006; Landgren et al., 2011; Deschaine et al., 2021), where they, directly and indirectly, regulate VTA dopamine activity and dopamine release [for review, see (Cornejo et al., 2021)]. Furthermore, GHSR-1A can form heterodimer receptor complexes with dopamine receptors and thus directly enhance the effect of dopamine receptors (Kern et al., 2012, 2015; Mustafa et al., 2021). Therefore, up-coming studies should explore the interaction between addiction, reward areas and heterodimerization of GHSR-1A and other receptors.

The GHSR-1A stands out as an attractive target for novel treatments for AUD and SUD. Due to GHSR-1As strong intrinsic activity, inverse agonists may have superior efficacy compared to conventional antagonists and has been proposed as the most attractive approach to treating AUD and SUD (Leggio, 2010). PF-5190457 is the first orally bioavailable GHSR-1A inverse agonist to proceed into clinical development (Bhattacharya et al., 2014). In the initial phase 1a/b trial, it appeared well-tolerated in both healthy individuals and AUD patients (Denney et al., 2017; Lee et al., 2020a,b) and reduced self-reported alcohol cue-elicited craving in a bar-like environment (Lee et al., 2020b). However promising, the small study population should be noted, as well as the fact that only one female was included. Nevertheless, current treatment options for AUD and SUD are few and often ineffective, and extensive clinical studies of GHSR-1A inverse agonist in addiction are warranted.

As such, data presented in this overview provide strong evidence for the GHSR-1A as a novel target for treatment. However promising, it is important to consider possible adverse effects of treatment that aims to inhibit GHSR-1A. With the key role ghrelin has with regards to homeostatic feeding and glucose metabolism (Tschop et al., 2000; Gray et al., 2019), metabolic changes and weight less is to be expected from such a treatment. Beyond ghrelin's traditional role, a recent study showed that the intrinsic activity of GHSR-1A is important for AMPA receptor trafficking and memory formation (Ribeiro et al., 2021). Consequently, an inverse agonist of GHSR-1A, such as PF-5190457, may cause cognitive deficits associated with learning. Furthermore, ghrelin and GHSR-1A have been proposed to have a significant role in the regulation of stress, as well as in anxiety and depression (for review, see Fritz et al., 2020), although much is left to be unraveled in this regard. Thus, possible adverse effects of manipulating GHSR-1A signaling must be carefully explored and considered in order to provide a safe treatment for AUD and SUD.

Nevertheless, the future possibilities and implications for ghrelin in addiction may go beyond that of treatment and could help find biomarkers for AUD and SUD. Naturally, drug exposure and taking are key for addiction development. Although this is a complex interaction of social, environmental, and neurobiological mechanisms, ghrelin's effect should not be disregarded. Interestingly, ghrelin appears to substantially increase novelty-seeking in both rats and humans, which may cause certain individuals to be more prone to drug-taking (Hansson et al., 2012). This fact, together with the association of plasma levels of ghrelin and genetic variations in genes related to ghrelin to AUD and SUD, raises a possibility for identifying high-risk individuals using components of the ghrelin system as possible biomarkers. Additionally, it has been shown that greater alcohol reward may predict the development of AUD in the future (King et al., 2014). This fact further strengthens the possibility for the ghrelin system to act as a predictive biomarker since the effect of ghrelin and GHSR on reward, in particular to alcohol, have been repeatedly demonstrated.

Furthermore, ghrelin may also be used as a way of monitoring AUD progress, withdrawal, relapse risk and treatment response. In fact, plasma ghrelin was recently proposed as a way of monitoring treatment response in AUD patients receiving baclofen (Geisel et al., 2019). Although the observations in plasma-association studies have thus far been unclear, and the role of circulating ghrelin in AUD and SUD is yet to be fully unraveled, there are a few points that need to be considered. Firstly, some, mainly earlier, plasma-association studies measured only total ghrelin and did not consider active acylated ghrelin. As previously mentioned, studies have shown associations between AUD and acylated, rather than total ghrelin (Koopmann et al., 2012, 2019; Sha et al., 2021). Secondly, the so-called inactive form of ghrelin, des-acyl-ghrelin, may have other functions than just being inactive and thus have its own implications for addiction (Delhanty et al., 2014; Ardeshiripur et al., 2018). Lastly, the relatively recent findings show that LEAP2 also acts on the GHSR as an inverse agonist and appears to attenuate the effects of ghrelin and impair dopaminergic signaling (Ge et al., 2018; M'kadmi et al., 2019; Islam et al., 2020; Mustafa et al., 2021). Although the role of DAG and LEAP2 for addiction is yet to be explored, it increases the depth of the ghrelin system and offers the possibility of exciting novel interactions between these three hormones and the reward system. Thus, future plasma-association studies should consider not only ghrelin but DAG and LEAP2 as well and are warranted to determine the relationship between the ghrelin system and addiction. Excitingly, these tools may in the future make it possible to identify high-risk individuals, to aid in the assessment of patients and to monitor and guide treatment for addiction disorders.

In conclusion, understanding the relationship between the ghrelin system and the development and maintenance of addiction is essential and must be further explored as it holds tremendous potential.

## Limitations With the Present Overview

Although the present overview summarizes the influence of GLP-1, amylin and ghrelin in addiction processes, it should be mentioned that other appetite-regulatory peptides have similar properties and may be promising targets for treatments of addictions as well (for review see Engel and Jerlhag, 2014). In brief, either activation of the leptin pathway or inhibition of orexin signaling reduces the motivation to consume alcohol and the intake thereof as well as stimulatory behaviors associated with other addictive drugs. Besides, suppression of neuropeptide Y2 receptors or cholecystokinin pathway reduces alcohol-related behaviors. Another appetite-regulatory peptide mediating alcohol intake is galanin. The lack of more detailed information on these in the present overview should be considered as a limitation.

This overview includes the most central information for each of the three peptides on behaviors of alcohol and drugs of abuse, without a detailed description of the methods, design, results or limitations for each article. This was done in an attempt not to overwhelm the reader with too much information. Therefore, to gain more detailed insight into this, the reader is guided to the original article. Moreover, the present overview summarizes the most relevant studies on GLP-1, amylin and ghrelin in addiction processes. Although the articles presented herein were not selected using PICOS, PRISM, Cochrane or JBI as they are for a systemic review, they were selected by means of keywords and free text words, as well as by multiple databases. In addition, a brief quality check of the design/results of selected articles was conducted. As a systematic review provides the highest levels of evidence and reliability, the lack of such review design of the present overview should be considered as a limitation.

## General Conclusion

As summarized above, upcoming studies should better define the neurocircuits and possible sex differences for each of the appetite-regulatory peptides as well as their modulation of addiction. These preclinical studies further reveal that GHSR-1A antagonists, GLP-R agonists or AMYR agonists individually prevent the acute rewarding properties of alcohol and other drugs. Besides, in chronic alcohol/drug models, they each reduce alcohol/drug intake, relapse and the motivation to consume alcohol and other drugs. However, it is unknown which of these appetite-regulatory peptides has the most profound effect on such behaviors or is associated with the least adverse effects. This is an intriguing question for additional studies doing systematical comparisons.

Social support networks are enormously beneficial for successful treatment of all addictive disorders. Recently, a protective social effect against addiction-like behavior was shown in rodent models (reviewed in Venniro et al., 2020) similarly to what is seen in humans. Experiments that evaluate drug intake in the context of choice between multiple rewards are more closely aligned with the reality of the human trajectory from recreational use to chronic drug use. In fact, in rodent models, the greater proportion of subjects tends to prefer non-drug rewards such as social or palatable food rewards, mimicking the prevalence of AUD/SUD in society. To be successful in treating human AUD/SUD, a treatment needs to target the population of individuals that continue to prefer drug reward over non-drug reward, especially when the drug reward is additionally associated with adverse events, such as pairing the drug reward with foot shocks or contaminating with bitter-tasting quinine. Additional studies should explore the effect of each individual system on choice when exposed to multiple rewards such as drugs, palatable food and social reward. In fact, for GLP-1 there are studies showing that Ex-4 skews the preference for certain types of food when multiple options are presented (Lopez-Ferreras et al., 2018), but alcohol and other drugs options have not been compared to non-drug options until now. This would provide additional information on subtypes of patients with addiction who might benefit from such treatments.

Another interesting perspective would be to evaluate the effect of a combination treatment of appetite-regulatory peptides on alcohol-mediated behaviors. This possible synergistic effect, associated with fewer side effects and tolerance development, would be beneficial in a clinical situation as the combination of sCT and liraglutide, for example, causes a sustained and additive weight loss in obese rats (Liberini et al., 2019). In AUD patients, the combination of pharmacological treatment and social adjustments has beneficial effects on sustained abstinence. Thus, the possibility that intervention with an appetite-regulatory peptide together with an enhanced social environment causes an advantageous reduction in alcohol intake in rodents should be explored.

To date more knowledge on the effect of different diets on the circulating levels of appetite-regulatory peptides are available. The findings that binge intake of high-fat diet alters the intake of palatable foods and blunts the response of appetite-regulatory peptides on hedonic feeding (Arcego et al., 2020), provides further evidence that appetite-regulatory peptides influence reward. Supportively, ketogenic diet decreases alcohol withdrawal symptoms in humans and reduces alcohol intake in rodents (Wiers et al., 2021). Therefore, upcoming studies should evaluate the effects of different diets and intake patterns (such as binge) on behaviors induced by alcohol or addictive drugs.

## Data Availability Statement

The original contributions presented in the study are included in the article/supplementary material, further inquiries can be directed to the corresponding author/s.

## Author Contributions

OS, MT-A, and EJ have all contributed to the present review, which was written together as a team.

## Funding

EJ was supported by grants from the Swedish Research Council (2019-01676), Arvid Carlsson foundation, The Swedish brain foundation, and LUA/ALF (Grant No. 148251) from the Sahlgrenska University Hospital. The funding source had no involvement in data collection, analysis and interpretation of data, in the writing of the report; and in the decision to submit the article for publication.

## Conflict of Interest

The authors declare that the research was conducted in the absence of any commercial or financial relationships that could be construed as a potential conflict of interest.

## Publisher's Note

All claims expressed in this article are solely those of the authors and do not necessarily represent those of their affiliated organizations, or those of the publisher, the editors and the reviewers. Any product that may be evaluated in this article, or claim that may be made by its manufacturer, is not guaranteed or endorsed by the publisher.
